# Influence of birth weight on calcaneal bone stiffness in Belgian pre-adolescent children

**DOI:** 10.1186/2049-3258-72-S1-P5

**Published:** 2014-06-06

**Authors:** Karen Van den Bussche, Nathalie Michels, Luis Gracia-Marco, Diana Herrmann, Gabriele Eiben, Stefaan De Henauw, Isabelle Sioen

**Affiliations:** 1 Department of Public Health, Ghent University, 9000 Ghent, Belgium; 2 GENUD (Growth, Exercise, NUtrition and Development) Research Group, University of Zaragoza, Zaragoza, Spain; 3 School of Sport and Health Sciences, University of Exeter, Exeter, UK; 4 BIPS - Institute for Epidemiology and Prevention Research GmbH, Bremen, Germany; 5 Department of Pediatrics, Institute of Clinical Sciences, The Queen Silvia Children’s Hospital, Sahlgrenska Academy at University of Gothenburg, Göteborg, Sweden; 6 Department of Health Sciences, Vesalius, University College Ghent, Ghent, Belgium; 7 FWO, Research Foundation Flanders, Egmontstraat 5, 1000 Brussels, Belgium

## Background

Several studies have shown associations between birth weight and adult bone mass. However, it is uncertain whether that influence of birth weight is already visible in childhood. This study investigated the relation between birth weight and calcaneal bone stiffness in a large sample of Belgian healthy pre-adolescent children.

## Materials and methods

Participants were 827 children (3.6–11.2 y, 51.6% boys) from the Belgian cohort of the IDEFICS study. Birth weight was obtained using a parental questionnaire and quantitative ultrasound (QUS) measurements were performed to determine the calcaneal Broadband Ultrasound Attenuation (BUA), Speed of Sound (SOS) and Stiffness Index (SI) using Lunar Achilles Device.

## Results

The average birth weight was 3435.7 ± 512.0 g for boys and 3256.9 ± 471.1 g for girls. The average calcaneal QUS measurements were equal to 89.6 ± 24.0 (23.3 to 153.9) dB/MHz for BUA, 1621.4 ± 49.6 (1516.3 to 1776.5) m/sec for SOS and 92.8 ± 15.6 (49.0 to 163.0) for SI. Birth weight was positively associated with BUA (r = 0.13; p = 0.002) and with SOS (r = -0.16; p < 0.001). The associations remained after correcting for age and sex in multiple regression analyses, but disappeared after correcting for anthropometric covariates.

## Conclusions

Our findings suggest that birth weight, as a rough proxy indicator for genetic and environmental influences during intrauterine life, is associated with BUA and SOS in pre-adolescent children and may therefore influence the risk of osteoporosis later in life. Further studies using QUS are needed to investigate the consistency of the results of this study.

